# Reducible Nonunited Type II Odontoid Fracture with Atlantoaxial Instability: Outcomes of Two Different Fixation Techniques

**DOI:** 10.3390/ijerph18157990

**Published:** 2021-07-28

**Authors:** Torphong Bunmaprasert, Vorapop Trirattanapikul, Nantawit Sugandhavesa, Areerak Phanphaisarn, Wongthawat Liawrungrueang, Phichayut Phinyo

**Affiliations:** 1 Department of Orthopaedics, Faculty of Medicine, Chiang Mai University, Chiang Mai 50200, Thailand; torpong197@gmail.com (T.B.); clap_tan@hotmail.com (V.T.); nantawitsu@gmail.com (N.S.); aphanphaisarn@hotmail.com (A.P.); mint11871@hotmail.com (W.L.); 2 Department of Family Medicine, Faculty of Medicine, Chiang Mai University, Chiang Mai 50200, Thailand; 3 Center for Clinical Epidemiology and Clinical Statistics, Faculty of Medicine, Chiang Mai University, Chiang Mai 50200, Thailand; 4 Musculoskeletal Science and Translational Research (MSTR) Cluster, Chiang Mai University, Chiang Mai 50200, Thailand

**Keywords:** atlantoaxial instability, nonunited odontoid fracture, posterior atlantoaxial fusion

## Abstract

Displaced nonunited type II odontoid fracture can result in atlantoaxial instability, causing delayed cervical myelopathy. Both Magerl’s C1-C2 transarticular screw fixation technique and Harms-Goel C1-C2 screw-rod segmental fixation technique are effective techniques to provide stability. This study aimed to demonstrate the results of two surgical fixation techniques for the treatment of reducible nonunited type II odontoid fracture with atlantoaxial instability. Medical records of patients with reducible nonunited type II odontoid fracture hospitalized for spinal fusion between April 2007 and April 2018 were reviewed. For each patient, specific surgical fixation, either Magerl’s C1-C2 transarticular screw fixation technique augmented with supplemental wiring or Harms-Goel C1-C2 screw-rod fixation technique, was performed according to our management protocol. We reported the fusion rate, fusion period, and complications for each technique. Of 21 patients, 10 patients were treated with Magerl’s C1-C2 transarticular screw fixation technique augmented with supplemental wiring, and 11 were treated with Harms-Goel C1-C2 screw-rod fixation technique. The bony fusion rate was 100% in both groups. The mean time to fusion was 69.7 (95%CI 53.1, 86.3) days in Magerl’s C1-C2 transarticular screw fixation technique and 75.2 (95%CI 51.8, 98.6) days in Harms-Goel C1-C2 screw-rod fixation technique. No severe complications were observed in either group. Displaced reducible, nonunited type II odontoid fracture with cervical myelopathy should be treated by surgery. Both fixation techniques promote bony fusion and provide substantial construct stability.

## 1. Introduction

Odontoid fractures account for approximately 7–13% of all cervical spinal fractures [1,2]. Anderson and D’Alonzo introduced a classification system in 1974 that had three subtypes according to the location of the fracture line [3]. The type II odontoid fracture is clinically important because fracture through the waist (fracture line occurs between the junction of the odontoid process and the body of the axis) relates to a high nonunion rate due to interruption of blood supply and its own instability [4]. Sometimes, clinical manifestations of type II odontoid fracture are not always immediately identifiable, and missed diagnosis often occurs due to poor visualization of plain radiography [2]. The delayed or untreated acute fracture may result in nonunion and subsequent atlantoaxial instability causing late cervical myelopathy [5]. Halo-vest immobilization was the optional treatment for acute type II odontoid fracture. However, according to the previous study, rigid collar and halo-vest immobilization provided a relatively high nonunion rate at 32% [6,7].

Instability of the atlantoaxial (C1-C2) joint may be the result of various pathologic processes, including degeneration, infection, inflammation, tumor, and trauma [8]. Displaced nonunited type II odontoid fracture is one of the common etiologies. It often leads to atlantoaxial instability, subsequent subluxation of the joint, and disabling neurological deficits [1,2,5]. In these cases, surgical treatment is the current standard treatment [2]. The goals of surgical treatment are to restore the alignment, decompress the neural structure and create rigid stability by performing fusion and fixation of C1-C2 vertebrae [7,9,10,11]. The surgical techniques of C1-C2 fixation, including Magerl’s C1-C2 transarticular screw fixation technique and Harms-Goel C1-C2 screw-rod segmental fixation technique, are well known among spine surgeons [9,10]. Both techniques provide strong biomechanical stability and higher fusion rates when compared to the posterior sublaminar wiring alone [11,12,13]. To further increase fixation stability and improve the union rate, an alternative technique to increase fixation stability by combining the transarticular screw augmentation with posterior supplemental wiring and fusion was proposed [14,15,16]. Previously, several studies had addressed the biomechanical properties and treatment outcomes for each fixation technique in patients with atlantoaxial instability [11]. To date, only a few studies and case reports have addressed the surgical outcomes of these fixation techniques in patients with atlantoaxial instability secondary to nonunited type II odontoid fracture.

This study aims to report the results of Magerl’s C1-C2 transarticular screw fixation technique and Harms-Goel C1-C2 screw-rod segmental fixation technique for the treatment of patients with nonunited type II odontoid fracture with atlantoaxial instability. We reported the treatment outcomes, in terms of union rate, the mean time to bony fusion, and complications of these two techniques in the group of patients who develop atlantoaxial instability and neurologic deficits from reducible nonunited type II odontoid fracture.

## 2. Materials and Methods

### 2.1. Study Design

We retrospectively reviewed 21 patients with nonunited type II odontoid fracture with atlantoaxial instability who had undergone surgical fusion at Chiang Mai University Hospital between April 2007 and April 2018. The Institutional Ethics Committee of the Faculty of Medicine, Chiang Mai University approved the study protocol (ORT-2559-04151). Informed consent of the patients was waived due to retrospective data collection. All patient’s personal information remained confidential. There was no cost, payment made, or harm to the patients as a result of this study.

### 2.2. Study Population

The inclusion criteria were patients with reducible, nonunited type II odontoid fracture with atlantoaxial instability treated with posterior atlantoaxial fusion surgery during the study period. The patient’s clinical presentation and symptoms were varied and included chronic upper neck or suboccipital pain, limitation of neck motion, spasticity, numbness, and weakness of the upper and lower extremities. Indications for surgery were progressive neurological deficit, deformity, or pain that did not improve from conservative treatment. The exclusion criteria were patients whose atlantoaxial instability resulted from degenerative processes, tumor, infection, inflammatory disorders, congenital diseases, and irreducible cases. Additionally, the alignment of the C1-C2 vertebrae must have been reduced by applying preoperative cervical traction in all cases to evaluate the possibilities of reduction during surgery in both techniques.

### 2.3. Surgical Fixation Techniques

**Figure 1:**
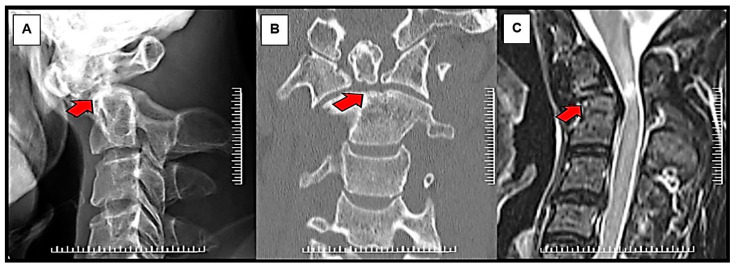
Radiographic images of nonunited type II odontoid fracture with atlantoaxial instability. Atlantoaxial instability with cervical myelopathy caused by nonunited type II odontoid fracture (Red arrows): (**A**) lateral plain radiograph; (**B**) coronal CT image; (**C**) sagittal MR image.

**Figure 2:**
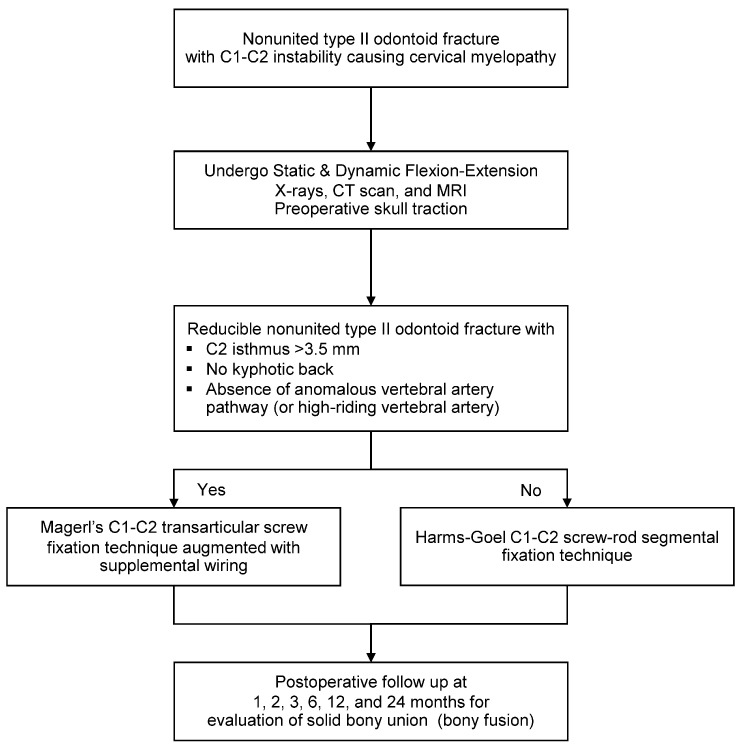
Patient management protocol at Chiang Mai University Hospital.

Before surgery, all patients had a standard series of radiographs, including the open mouth, anterior-posterior, static lateral, and dynamic lateral flexion/extension views. The lateral flexion-extension view was used to evaluate the instability of the C1-C2 complex, including the possibility of reduction that could be achieved intraoperatively. Excessive C1-C2 translation and lateral atlantodental interval more than 3 mm generally caused instability. Thus, in this case, the fusion of C1-C2 joints should be carried out. Computed tomography (CT) scan and magnetic resonance imaging (MRI) were also performed to evaluate further the bony anatomy detail of the upper cervical spine, the spinal cord, and the vertebral artery status (Figure 1). In our institution, the type of surgical fixation, either Magerl’s C1-C2 transarticular screw fixation technique augmented with supplemental wiring or Harms-Goel C1-C2 screw-rod segmental fixation technique, was selected based on local management protocol, which considered the width of the C2 isthmus, the vertebral artery pathway, and the presence or absence of a kyphotic back. Magerl’s C1-C2 transarticular screw fixation technique would be chosen for patients with a C2 isthmus wider than 3.5 mm, absence of anomalous vertebral artery pathway, and no kyphotic back. Harms-Goel C1-C2 screw-rod segmental fixation technique was performed in the rest of the patients who were not indicated for Magerl’s C1-C2 transarticular screw fixation technique (Figure 2).

Intraoperatively, the patients were placed in the prone position with skull immobilization. Cervical alignment was reduced and checked using intraoperative fluoroscopy. The perfect reduction was not attempted in this step because the full extension position could potentially obstruct wire passage under the posterior arch of C1. A posterior midline longitudinal incision was made from a few centimeters below the external occipital protuberance to the spinous process of C3. Dissection through the midline avascular raphe reduced bleeding from the paravertebral musculature. The posterior arch of C1 and the posterior element of C2 were then exposed. A small absorbable suture was passed beneath the posterior arch of C1 in the cranial-to-caudal direction. This suture was used to perform further reduction to complete the acceptable alignment and counteract the pushing force during screw insertion. Alternatively, a reduction hook was used to partially compress the posterior arch of C1 to C2 to reduce local kyphosis. This step facilitated us to achieve the perfect reduction.

#### 2.3.1. Magerl’s C1-C2 Transarticular Screw Fixation Technique Augmented with Supplemental Wiring

The medial border of the C2 pedicles and pars interarticularis were identified, then a 4.5 mm standard cortical screw was inserted from the C2 pars interarticularis, past the C1-C2 joints, and directed to the anterior arch of C1 and into the C1 lateral masses. The screw trajectory, screw length, and insertion were guided by a lateral image intensifier. In most cases, the Magerl’s C1-C2 transarticular screws were augmented with posterior supplemental wiring. Decortication of the posterior elements of C1 and C2 was crucial for fusion success. An autogenous corticocancellous bone graft was harvested from the posterior iliac crest. The structural bone graft was placed over the fusion site and secured with wire under optimal tension.

#### 2.3.2. Harms-Goel C1-C2 Screw-Rod Segmental Fixation Technique

The entry point for the C1 lateral mass screw was marked using a 3 mm high-speed burr. A pilot hole, reaching the anterior cortical bone, was then made using a 2.7 mm drill. This was followed by a straight or slightly convergent trajectory in the posterior-anterior direction parallel to the plane of the C1 posterior arch in the sagittal direction. The hole was tapped through the entire lateral masses. Screws of 4.0 mm diameter and 28 mm to 34 mm in length with polyaxial heads were inserted bicortically into the lateral mass of C1. Subsequently, the pedicles of the C2 were identified. The entry point for the placement of the C2 pedicle screw was marked with a drill at a point 2 mm from the medial border and 5 mm from the caudal border of the C2 articular process. Under C-arm guidance in the lateral projection, a hole was drilled parallel to the screws inserted in C1 at an angle of 20° to 30° cranially up to and through the anterior cortical bone. In the transverse plane, the screws were placed in a convergent direction at an angle of 20° to 25°. The screws 4.0 mm in diameter and 24 mm to 32 mm in length were then inserted. Two 3.2 mm connecting rods were applied and then attached to the screws. Autogenous corticocancellous bone graft was harvested from the posterior iliac crest; then, the structural bone graft was placed over the fusion site.

**Figure 3:**
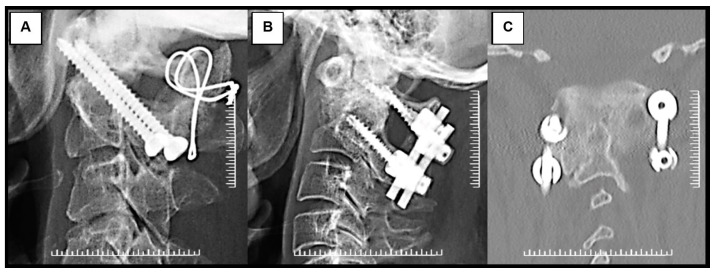
Radiographic images of patients with reducible nonunited type II odontoid fracture with myelopathy treated with (**A**) Magerl’s C1-C2 transarticular screw fixation technique augmented with supplemental wiring and (**B**) Harms-Goel C1-C2 screw-rod segmental fixation technique. Coronal CT scan imaging (**C**) shows C1-C2 complex screw construction with solid fusion mass in Harms-Goel C1-C2 screw-rod segmental fixation technique at 3-month follow-up.

Structural and morselized autogenous bone graft was added in all cases in this series. All patients were allowed to ambulate a few days after surgery, after which their neurological status was reevaluated. Rigid cervical orthosis was utilized for 8 to 12 weeks until the solid bony union was observed. Patients in both groups were followed up in terms of clinical improvement and radiographic results at 1, 2, 3, 6, 12, and 24 months after surgery. Plain radiographs (open mouth view, anterior-posterior, lateral flexion, and extension radiographs) and CT scans were made three months after surgery to evaluate fusion (Figure 3).

### 2.4. Study Endpoints

The primary study endpoints were bony fusion rate at 90, 120, and 180 days and mean time to bony fusion. Solid bony fusion was defined as the presence of trabecular continuity through the posterior arch of C1 and C2 and less than 2 mm of motion between the segments in flexion-extension views. Secondary study endpoints were operative time, intraoperative blood loss, length of stay after surgery, cost of hospital stays, postoperative Frankel’s score at two time-points (at postoperation and the latest follow-up).

### 2.5. Statistical Analysis

All statistical analyses were performed using Stata 16 (StataCorp, College Station, TX, USA). For continuous variables with normal distribution, mean and standard deviation were used for the data description, whereas the median and interquartile range were used for continuous variables with non-normal distribution. Frequency and percentages were used for the description of categorical variables.

For primary endpoints, the fusion rate in each group at 90, 120, and 180 days was estimated with Kaplan–Meier methods. We reported the fusion probability at each time point with its corresponding confidence interval. Restricted mean time to fusion in each group was calculated using flexible parametric survival regression described by Royston et al. [17]. Descriptive statistics were used for the presentation of secondary endpoints. As patients were allocated in each treatment group based on absolute indication and contraindication of Magerl’s C1-C2 transarticular screw fixation technique, patients in both groups were not within the same domain and were not comparable. Therefore, a statistical comparison was not made in this study.

## 3. Results

During the 10-year period, this study included 19 men and 2 women with an average age of 44.48 years (range 11–69 years). There were 10 patients in the Magerl’s C1-C2 transarticular screw fixation technique group and 11 patients in the Harms-Goel C1-C2 screw-rod segmental fixation technique group. No patients received occipital-cervical (O-C) fusion in our study. Baseline clinical characteristics, mechanism of injury, fracture characteristics, and preoperative Frankel’s score of the patients in each treatment group are shown in Table 1.

**Figure 4:**
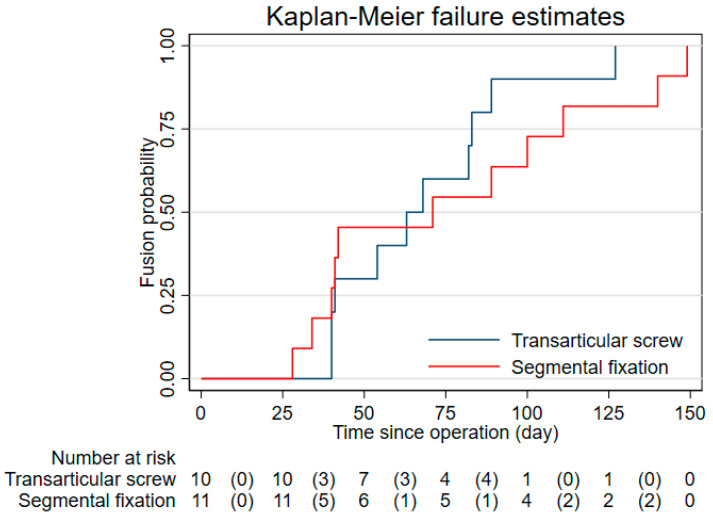
Kaplan-Meier curves for fusion probability in both study groups. The blue line depicted the fusion probability of patients in Magerl’s C1-C2 transarticular screw fixation technique group. The red line depicted the fusion probability of patients in the Harms-Goel C1-C2 screw-rod segmental fixation technique group.

The solid bony fusion rate at 90, 120, and 180 days were 90.0, 90.0, 100 in the Magerl’s C1-C2 transarticular screw fixation technique group and 63.6, 81.8, 100.0 in the Harms-Goel C1-C2 screw-rod segmental fixation technique group (Figure 4). No loosening or breakage of internal fixation occurred in either group. The mean time to fusion period was 69.7 (95%CI 53.1, 86.3) days in the Magerl’s C1-C2 transarticular screw fixation technique group and 75.2 (95%CI 51.8, 98.6) days in the Harms-Goel C1-C2 screw-rod segmental fixation technique group. The latest bony fusion was documented at 127 and 149 days from operation in the Magerl’s C1-C2 transarticular screw fixation technique group and the Harms-Goel C1-C2 screw-rod segmental fixation technique group, respectively.

Mean operative time was 152.5 ± 45.0 min in the Magerl’s C1-C2 transarticular screw fixation technique augmented with supplemental wiring group and 146.2 ± 28.7 min in the Harms-Goel C1-C2 screw-rod segmental fixation technique group. The median volume of intraoperative blood, the length of hospital stays after surgery, the overall costs during hospital stays including surgical implants, and postoperative Frankel’s score are shown in Table 2.

No severe complications (e.g., iatrogenic vertebral artery or spinal cord injury) were observed in either group. One patient had occipital neuralgia in the Magerl’s C1-C2 transarticular screw fixation technique group that improved at the 1-year follow-up. In the Harms-Goel C1-C2 screw-rod segmental fixation technique group, one patient experienced wound infection at the donor site of the iliac bone graft. However, the clinical improvement was observed after only a few days following the administration of intravenous antibiotics.

## 4. Discussion

Type II odontoid fracture concomitant with atlantoaxial instability is the complex injury of the upper cervical spine. The natural healing process of the fracture site is usually ineffective, and there is a higher rate of nonunion, which often leads to fracture displacement and compression of the spinal cord or nerve roots, causing delayed cervical myelopathy [18]. Surgery for cervical myelopathy in neglected displaced nonunited type II odontoid fractures is necessary when the patient develops progressive neurological deficits [2,18]. In those cases, posterior atlantoaxial fusion is the treatment. In this study, we reported the clinical outcomes in terms of bony fusion rate and time to the fusion of two surgical fixation techniques used in patients with nonunited type II odontoid fracture with atlantoaxial instability, Magerl’s C1-C2 transarticular screw fixation technique augmented with supplemental wiring and Harms-Goel C1-C2 screw-rod segmental fixation technique. Both fixation techniques result in bony fusion prior to 180 days after surgery without any severe surgical complications.

Various techniques for posterior C1-C2 fusion have been described. Modern C1-C2 instrumentation techniques, including Magerl’s C1-C2 transarticular screw fixation technique augmented with supplemental wiring and Harms-Goel C1-C2 screw-rod segmental fixation technique, are effective in creating stability [9,10,11]. Both techniques were analyzed from cadaveric biomechanical studies and have been shown that they provided significant stabilization of the C1-C2 complex in all axis of rotation [19]. Lee et al. reported the clinical and radiographic study comparing the results of both techniques in 55 patients with symptomatic atlantoaxial instability from various causes. They found an 82% solid fusion rate in the transarticular fixation group and 96% for the segmental C1 lateral mass–C2 pedicle fixation group [20]. Meta-analysis and literature review by Elliott et al. compared the outcomes of these two techniques in patients with atlantoaxial instability resulting from odontoid fracture, os odontoideum, rheumatoid arthritis, and other causes [11]. They concluded that both techniques were safe and effective treatment options for C1-C2 instability, whereas the screw–rod technique provided slightly higher rates of fusion and lesser risk of vertebral artery injury during screw placement. Overall, the Harms-Goel screw-rod segmental fixation technique seemed to be a stronger fixation method.

Thus far, no study has demonstrated the outcome of these two techniques in specific groups of patients with reducible nonunited type II odontoid fractures resulting in atlantoaxial instability. We hypothesized that surgical treatment of atlantoaxial instability resulting from a nonunited type II odontoid fracture might be somewhat different from the instability resulting from other causes, e.g., rheumatoid arthritis. Based on our results, we found that the two fixation techniques were similar in terms of fusion rate, time to fusion, operative time, and length of postoperative hospital stay. However, compared to the Harms–Goel technique, Magerl’s C1-C2 transarticular screw fixation technique showed less time to bony fusion, less blood loss, and lower cost. This was in contrast to the available evidence [20,21], revealing a higher fusion rate in the Harms–Goel technique. Differences in the fusion techniques, graft materials used, and postoperative management protocol might explain the discrepancy of results.

Intraoperative and postoperative complications have been previously reported, such as malposition of the screw, implant failure, pseudarthrosis, leakage of cerebrospinal fluid, occipital neuralgia, and vertebral artery injury [20]. Several studies had reported the effect of atlantoaxial fusion on the postoperative loss of cervical rotation and neck stiffness. In one study of thirty patients with odontoid fractures who underwent posterior atlantoaxial fusion, it was found that the mean physiological cervical ranges of motion were decreased in all planes after fusion [22]. Moreover, adjacent intervertebral disorders might occur after atlantoaxial articulations. Another study was conducted on 65 patients who underwent atlantoaxial fusion by either Magerl’s C1-C2 transarticular screw fixation technique and posterior wiring or Harms-Goel C1-C2 screw-rod segmental fixation techniques. It was found that when fusion was performed in a hyperlordotic position, there was a kyphotic sagittal alignment after surgery in both groups, which could lead to subsequent subaxial kyphosis [23]. Thus, in our practice, the reduction and fusion of the atlantoaxial joints were preferably performed in a neutral position to prevent this late complication. In this study, only two minor complications were documented in our study: occipital neuralgia and graft donor site superficial infection. Preoperative studies of the individual’s anatomy of the atlantoaxial joints and surrounding vital structures may avoid serious complications. Meticulous dissection, good intraoperative imaging, and surgical techniques also facilitate favorable outcomes.

Compared to the Harms-Goel C1-C2 screw-rod segmental fixation technique, Magerl’s C1-C2 transarticular screw fixation technique augmented with supplemental wiring represents a potentially cost-saving alternative. There are, nevertheless, many prerequisites to transarticular fixation surgery. The absence of a high-riding vertebral artery, a wide C2 isthmus, nonkyphotic back, and perfectly anatomical reducible C1-C2 instability must be demonstrated before proceeding to surgery [24,25]. On the other hand, the Harms-Goel technique can be performed in kyphotic patients [26,27]. It is also feasible to perform this type of fixation in patients with irreducible fractures [27]. In patients with irreducible atlantoaxial instability, an extension of surgical fusion to the occiput should be considered. However, it is important to note that with posterior O-C fusion, a longer period of postoperative rigid orthosis might be needed. This study focused only on patients with reducible atlantoaxial instability secondary to nonunited type II odontoid fracture for whom O-C fusion was usually avoided as it creates a substantial loss of cervical flexion–extension and rotation and increases the risk of nonunion and implant failure.

There were some limitations to be addressed. First, the data were retrospectively collected from the hospital database for each treatment group. Therefore, the verification of the study endpoint might be delayed in some patients with incomplete visits. However, this would only underestimate the time to bony fusion. Second, the data on postoperative loss of cervical range of motion were not routinely documented. Thus, we were unable to correctly estimate the effect of both fixation methods on a postoperative range of motion. Third, the study size was relatively small to provide a precise estimation of confidence intervals of fusion rate and fusion time and might not be enough to conclude the absence of serious surgical endpoints. A prospective study with a larger study size is warranted to properly determine the clinical effectiveness and safety of the two surgical fixation methods.

## 5. Conclusions

In conclusion, displaced nonunited type II odontoid fracture with myelopathy should be treated by surgical fixation, using either Magerl’s C1-C2 transarticular screw fixation technique or the Harms-Goel C1-C2 screw-rod segmental fixation technique. Preoperative clinical and fracture characteristics should be considered in choosing the appropriate surgical fixation method for the patients. In patients with nonunited type II odontoid fracture with atlantoaxial instability, both fixation techniques resulted in a complete bony fusion rate prior to six months after surgery without causing severe surgical complications.

## Figures and Tables

**Table 1:** Clinical and fracture characteristics of the patients.

Characteristics	Magerl’s C1-C2 Transarticular Screw Fixation Technique Group (*n* = 10)		Harms-Goel C1-C2 Screw-Rod Segmental Fixation Technique Group (*n* = 11)	
Age (year, mean ± SD)	46.0	±21.8	42.8	±15.9
Gender (Male: Female, %male)	9:1	(90.0)	10:1	(90.9)
Mechanism of injury (*n*, %)				
Falling	4	(40.0)	6	(54.6)
Motor vehicle accident	6	(60.0)	5	(45.5)
Fracture characteristics				
Translation (mm, mean ± SD)	6.3	±2.3	6.1	±3.2
Angulation	24.3	±6.5	24.2	±10.1
Direction (*n*, %)				
Anterior	9	(90.0)	8	(72.7)
Posterior	1	(10.0)	3	(27.3)
Duration of neck pain (month, median (IQR))	12	(6, 60)	5	(2, 36)
Duration of myelopathy (month, median (IQR))	2	(1.5, 3)	2	(1, 3)
Preoperative Frankel’s score (*n*, %)				
A	0	0	0	0
B	2	(20.0)	3	(27.3)
C	3	(30.0)	3	(27.3)
D	5	(50.0)	5	(45.4)
E	0	0	0	0

Abbreviations: IQR, interquartile range; SD, standard deviation.

**Table 2:** Primary and secondary endpoints.

Clinical Endpoints	Magerl’s C1-C2 Transarticular Screw Fixation Technique Group (*n* = 10)		Harms-Goel C1-C2 Screw–Rod Segmental Fixation Technique Group (*n* = 11)	
**Primary endpoints**				
Number of patients with bony fusion	10	(100)	11	(100)
Fusion rate (%)				
At 90 days (%, 95%CI)	90.0	(64.2, 99.4)	63.6	(37.3, 88.8)
At 120 days (%, 95%CI)	90.0	(64.2, 99.4)	81.8	(55.8, 97.2)
Mean time to fusion (days, 95%CI)	69.7	(53.1, 86.3)	75.2	(51.8, 98.6)
**Secondary endpoints**				
Operative time (minute, mean ± SD)	152.5	±45.0	146.2	±28.7
Intraoperative blood loss (ml, median (IQR))	150	(100, 200)	200	(100, 300)
Length of stay after surgery (day, mean ± SD)	9.5	(7, 17)	9	(5, 15)
Hospital stay cost (USD, median (IQR))	1882	(1528, 2096)	3775	(3132, 6262)
Postoperative Frankel’s score				
Initial postoperative (*n*, %)				
A	0	0	0	(0)
B	2	(20.0)	1	(9.0)
C	2	(20.0)	5	(45.5)
D	4	(40.0)	5	(45.5)
E	2	(20.0)	0	(0)
Latest follow-up (*n*, %)				
A	0	0	0	0
B	0	0	1	(9.0)
C	1	(10.0)	1	(9.0)
D	5	(50.0)	4	(36.5)
E	4	(40.0)	5	(45.5)

Abbreviations: CI, confidence interval; IQR, interquartile range; SD, standard deviation, USD, United States Dollar (currency rate 1 USD = 30.28 Thai Baht).

## Data Availability

The datasets used and/or analyzed during the current study are available from the corresponding author on reasonable request.
